# Pleiotrophin enhances PDGFB-induced gliomagenesis through increased proliferation of neural progenitor cells

**DOI:** 10.18632/oncotarget.12983

**Published:** 2016-10-28

**Authors:** Lei Zhang, Liisi Laaniste, Yiwen Jiang, Irina Alafuzoff, Lene Uhrbom, Anna Dimberg

**Affiliations:** ^1^ Department of Immunology, Genetics and Pathology, Science for Life Laboratory, Uppsala University, The Rudbeck Laboratory, Uppsala, Sweden; ^2^ Division of Brain Sciences, Imperial College Faculty of Medicine, London, UK; ^3^ Department of Molecular Biochemistry and Biophysics, Karolinska Institute, Solna, Sweden; ^4^ Department of Pathology, Uppsala University Hospital, Uppsala, Sweden

**Keywords:** glioma, pleiotrophin, gliomagenesis, tumor initiation, chromosome 7

## Abstract

Pleiotrophin (PTN) augments tumor growth by increasing proliferation of tumor cells and promoting vascular abnormalization, but its role in early gliomagenesis has not been evaluated. Through analysis of publically available datasets, we demonstrate that increased PTN mRNA expression is associated with amplification of chromosome 7, identified as one of the earliest steps in glioblastoma development. To elucidate the role of PTN in tumor initiation we employed the RCAS/*tv-a* model that allows glioma induction by RCAS-virus mediated expression of oncogenes in neural progenitor cells. Intracranial injection of RCAS-PTN did not induce glioma formation when administrated alone, but significantly enhanced RCAS-platelet derived growth factor (PDGF)B-induced gliomagenesis. PTN co-treatment augmented PDGFB-induced Akt activation in neural progenitor cells *in vitro*, and enhanced neural sphere size associated with increased proliferation. Our data indicates that PTN expression is associated with chromosome 7 gain, and that PTN enhances PDGFB-induced gliomagenesis by stimulating proliferation of neural progenitor cells.

## INTRODUCTION

Malignant glioma comprises a group of primary brain tumors with a general poor prognosis. The vast majority of gliomas are adult grade II-IV oligodendrogliomas or astrocytomas that grow invasively into the cortex [1]. Glioblastoma (GBM) is the most aggressive type of glioma, and can either arise de novo or progress from lower-grade gliomas. Based on transcriptome profiling, four molecular subtypes: proneural, mesenchymal, classical and neural have been identified in GBM [2]. Studies of the somatic genomic alterations reveal that classical tumors harbor high level of EGFR amplification, mesenchymal samples are associated with NF1 mutations, proneural with PDGFRA alterations or IDH1/2 mutations while the neural subtype lacks specific genetic abnormalities [3]. Proneural tumors that have IDH1/2 mutations are of the glioma-CpG island methylator phenotype (G-CIMP). Mathematical modeling and *in vivo* experiments have suggested that most human G-CIMP^−^ tumors may evolve from a common proneural-like glioma and indicated that gain of chromosome 7 and loss of chromosome 10 are common early events of gliomagenesis [4] PDGFA amplification was found to be the most likely initial driver of glioma formation, and sufficient for gliomagenesis in mice, but the potential contribution of other chromosome 7 genes to the initial oncogenic events are still unclear.

One of the genes located on chromosome 7 is pleiotrophin (*PTN*), encoding a heparin-binding cytokine. PTN binds to and inactivates receptor-type protein tyrosine phosphatase receptor ζ (PTPRζ), leading to increased phosphorylation of its substrates [5]. PTN also activates anaplastic lymphoma kinase (ALK) [6]. PTN is up-regulated in glioma, and its expression is associated with poor survival in astrocytomas and glioblastoma [7]. PTN can stimulate glioma cell migration and proliferation, and blocking PTN or its receptors reduces tumor growth [5, 6, 8]. Additionally, PTN can enhance glioma growth by promoting vascular abnormalization [7]. PTN has been implicated in maintaining the stemness of glioma initiating cells, but its role in early gliomagenesis has not been investigated [9].

Here, we show that gain of chromosome 7 in human gliomas is associated with up-regulation of PTN. By employing the RCAS/*tv-a* mouse model, we provide evidence that PTN is not sufficient to induce glioma development, but augments PDGFB-induced gliomagenesis by increasing Akt activation in neural progenitor cells.

## RESULTS

### PTN up-regulation is most prominent in the classical subgroup of gliomas and associates with chromosome 7 gain

**Figure 1:**
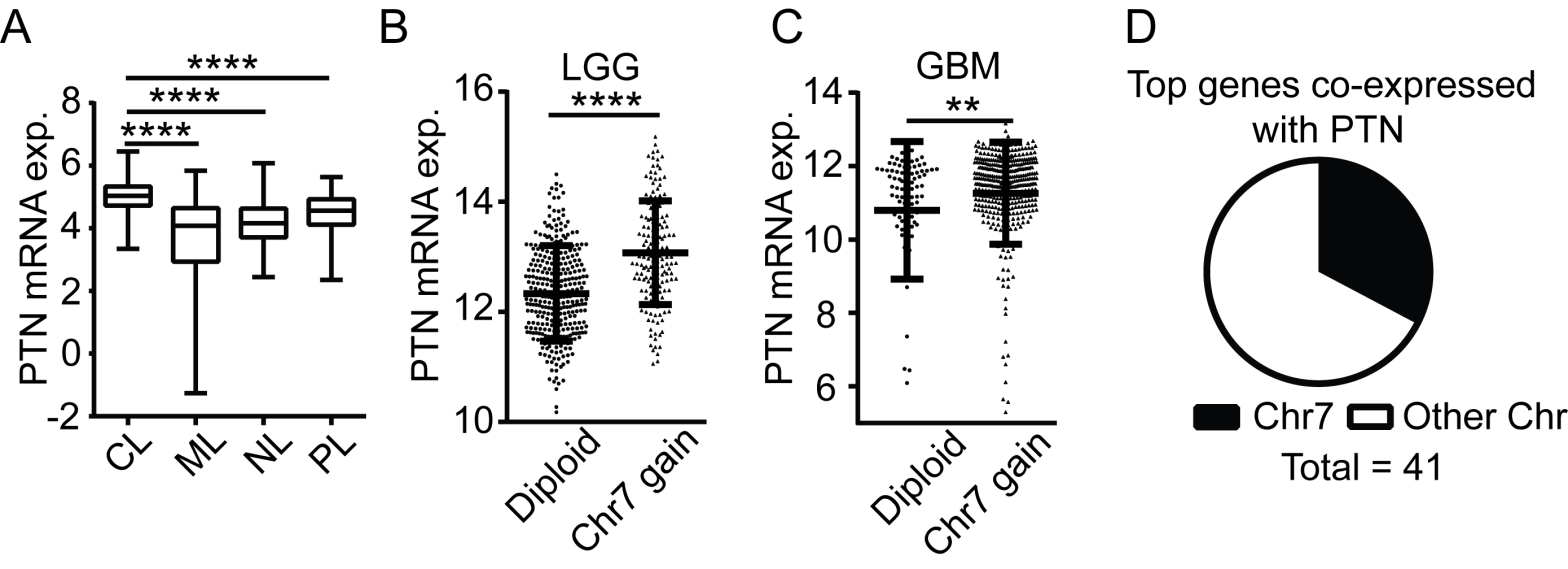
PTN up-regulation is associated with chromosome 7 gain **A.** PTN expression in tumors of different glioblastoma subtypes. The data is based on analysis of 114 classical (CL), 126 mesenchymal (ML), 65 neural (NL) and 80 pro-neural (PL) subtype glioblastomas. **B.**-**C.** PTN expression in chromosome 7 diploid (Diploid) or chromosome 7 gain (Chr7 gain) samples of lower-grade glioma (grade II and III, LGG) **B.** or glioblastoma (GBM) patients **C.**. **D.** Proportion of the top 41 PTN co-expressed genes located on chromosome 7 (Chr7) or other chromosomes (Other Chr).

To investigate the expression pattern of PTN in glioblastomas, we employed the Oncomine database which provides a systematic approach to analyze gene expression in publically available microarray datasets [10]. Differential analysis of gene expression of five independent datasets confirmed a consistent up-regulation of PTN mRNA in glioblastoma samples as compared with normal white matter (Figure S1A-S1E, Table S1) [11-14]. A meta-analysis of these datasets revealed that PTN is within the top 5% expressed genes in the GBM datasets. To determine if PTN overexpression is characteristic to a specific subtype of GBM, data was extracted from the cBioPortal database and cross-referenced with previously reported subtype information [2]. PTN expression was significantly higher in the classical subtype as compared to mesenchymal, pro-neural and neural tumors (Figure 1A).

The PTN gene is located on chromosome 7, which is most commonly subjected to broad amplification in GBM tumors of the classical subtype [2] [15]. We utilized datasets (TCGA LGG; TCGA GBM) from the GlioVis database to investigate if PTN expression occurs through chromosome 7 amplification. PTN mRNA expression was significantly higher in lower-grade glioma (LGG) and glioblastoma samples that showed a gain of chromosome 7 as compared to diploid tumors (Figure 1B-1C). Using Pearson's correlation analysis, we found that 5 out of the top 8 genes co-expressed with PTN in LGG and GBM are located on chromosome 7 (Table S2). Moreover, 14 of the 41 genes with a correlation coefficient of more than 0.61 are located on chromosome 7 (Figure 1D). Thus, increased PTN expression in glioma is associated with amplification of chromosome 7.

### PTN is not sufficient for gliomagenesis in the RCAS/*tv-a* model

**Figure 2:**
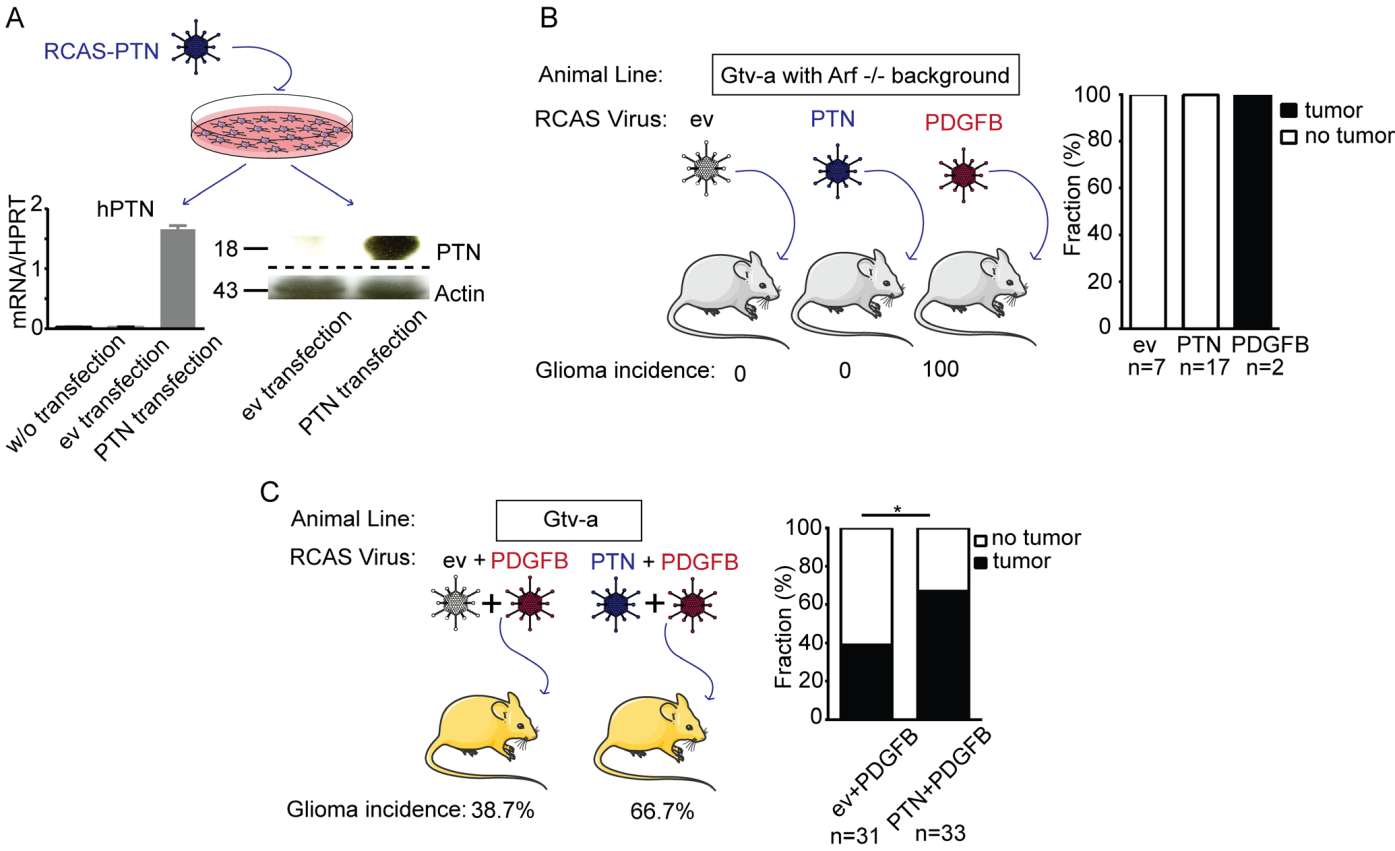
PTN over-expression does not induce glioma formation but enhances PDGF-B induced gliomagenesis in the RCAS/*tv-a* model of glioma **A.** qPCR and western blot analysis of *tv-a* expressing NPCs treated with virus-containing media from DF-1 cells transfected with RCAS-ev or RCAS-PTN. **B.** Glioma incidence in *Gtv-a;Arf* mice infected with RCAS-ev (empty vector, negative control), RCAS-PTN or RCAS-PDGFB (positive control) virus. **C.** Glioma incidence in *G/tv-a* wild type mice infected with RCAS-ev in combination with RCAS-PDGFB or RCAS-PTN in combination with RCAS-PDGFB.

Mathematical modeling of non-GCIMP glioblastoma subgroups demonstrated that gain of chromosome 7 and loss of chromosome 10 are likely to be early events in glioma formation [4]. We employed the RCAS/*tv-a* model [16] to determine the oncogenic potential of PTN during gliomagenesis. The human PTN (hPTN) gene was cloned into the RCAS vector and the construct was transfected into DF1 cells. PTN mRNA and protein expression was detected in primary murine brain neural progenitor cells (NPCs) treated with conditioned medium from DF1 RCAS-PTN cells, confirming efficient virus production (Figure 2A). Neonatal *G/tv-a;Arf^-/-^* mice, expressing the *tv-a* receptor under control of a GFAP-promoter, were intracranially injected with equal numbers of DF1 RCAS-ev, DF1 RCAS-PTN or DF1 RCAS-PDGFB. Consistent with previous studies, mice injected with the RCAS-PDGFB virus were afflicted with high-grade glioma within 4 weeks of infection [16]. However, the RCAS-PTN virus did not induce tumors up to 23 weeks after virus injection (Figure 2B). We conclude that increased PTN expression is not sufficient for brain tumor initiation in neonatal *Arf ^-/-^* mice.

### PTN enhances PDGF-B induced gliomagenesis in the RCAS/*tv-a* model

To analyze if PTN promotes PDGFB-induced gliomagenesis, neonatal *G/tv-a* wildtype mice were intracranially injected with DF1 RCAS-PDGFB in combination with DF1 RCAS-ev (RCAS-PDGFB+RCAS-ev), or DF1 RCAS-PDGFB in combination with DF1 RCAS-PTN (RCAS-PDGFB+RCAS-PTN). Tumor incidence, as determined by the presence of Ki-67^+^ cells, was strikingly increased in mice injected with RCAS-PDGFB+RCAS-PTN (66.7%) as compared to mice injected with RCAS-PDGFB+RCAS-ev (38.7%) (Fisher's exact test, *P* = 0.0467) (Figure 2C).

PDGFB cDNA was detected in all gliomas, and hPTN cDNA was present in all mice where RCAS-PDGFB and RCAS-PTN were combined (Figure S2A). We did not detect any tumors in co-injected mice that had lost either the PDGFB cDNA or the PTN cDNA construct, indicating that the combined expression of both genes enhanced tumor formation.

**Figure 3:**
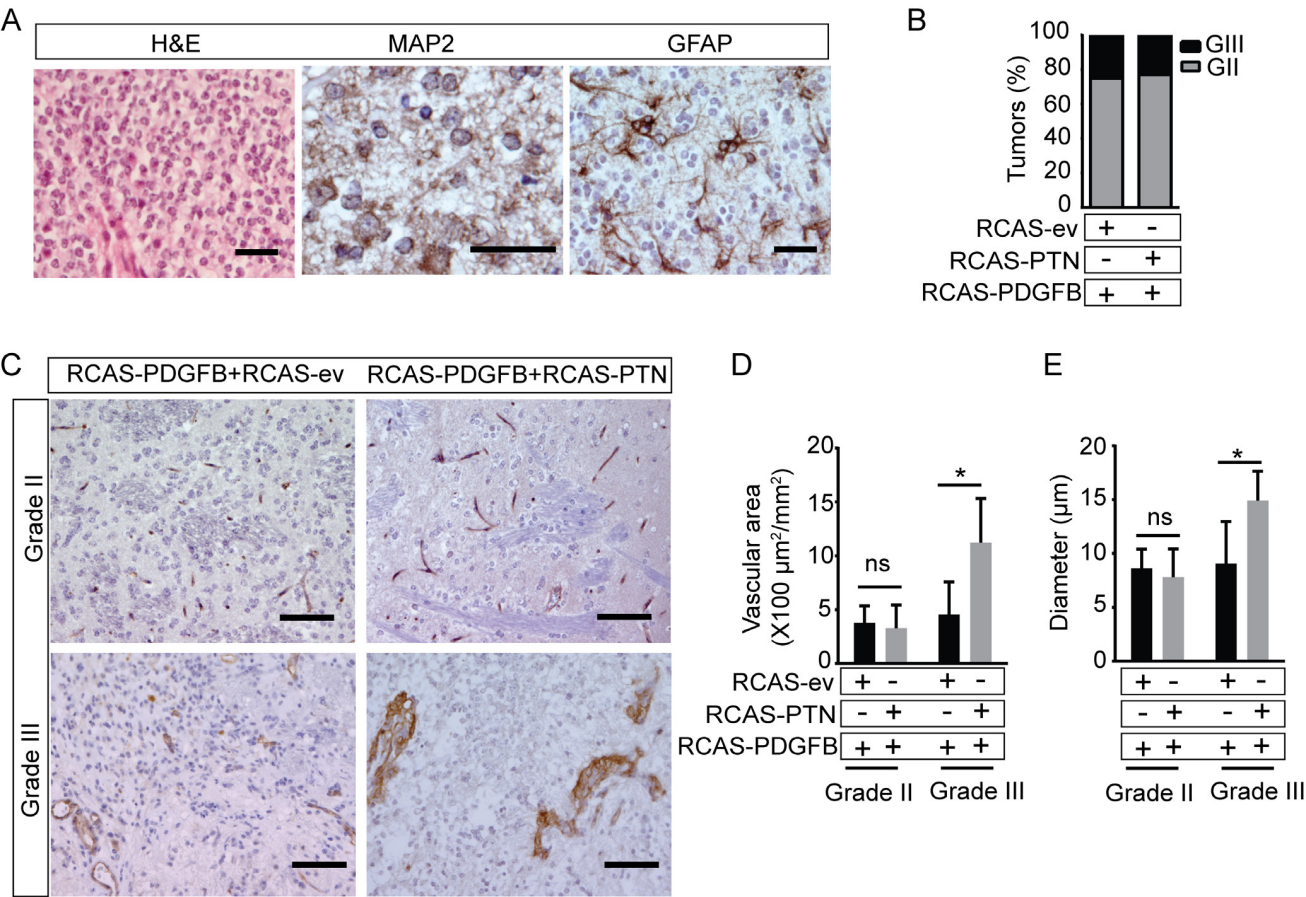
Co-infection with RCAS-PTN does not alter the histopathology of RCAS-PDGFB induced gliomas, but enhances angiogenesis and vascular abnormalization in high grade tumors **A.** H&E stain and immunohistochemical staining of MAP2 and GFAP. **B.** Distribution of pathological grades. Results were plotted as percentage of samples per grade in *G/tv-a* wt mice injected with the RCAS-PDGFB+RCAS-ev (*n* = 12) or RCAS-PDGFB+ RCAS-PTN (*n* = 22). (GII: grade II, GIII: grade III). **C.**-**E.** Immunohistochemical staining of CD31 and stereological quantification of vessel area and vessel diameter in RCAS-PDGFB+RCAS-ev and RCAS-PDGFB+RCAS-PTN grade II and grade III gliomas (means ± SD; *n* = 3-5 mice/group). (Bar = 20 μm in A, Bar = 100 μm in C)

To determine if PTN affected the histological subtype of PDGFB-induced tumors, we examined the histopathology of the brain tumors. PDGFB-expression induces tumors with features similar to human grade II diffuse oligodendroglioma or grade III anaplastic oligodendroglioma in *G/tv-a* wt mice [16]. The tumors grew diffusely and consisted of small tumor cells with regular round nuclei and perinuclear halo (Figure 3A). Tumor cells were positive for MAP2 with a cap-like staining pattern, characteristic of oligodendroglioma. Scattered GFAP-positive reactive astrocytes were found within and around the tumor tissue, but tumor cells were GFAP negative. The histopathology of gliomas induced by RCAS-PDGFB+RCAS-PTN was similar to gliomas induced by RCAS-PDGFB+RCAS-ev. Tumors were graded according to WHO diagnostic criteria [1]. Grade II tumors grew diffusely in the brain, a proportion of the cells were Ki-67^+^, and apoptotic cells were present (Figure S2B-C). Grade III tumors were defined as having at least two of the following features: mitotic figures (Figure S2D), microvascular proliferation (Figure S2E), cellular and nuclear pleomorphism and pseudopalisading necrosis (Figure S2F). A similar proportion of grade III and grade II tumors formed in mice induced by RCAS-PDGFB+RCAS-PTN as in mice induced by RCAS-PDGFB+RCAS-ev (Figure 3B). Taken together, co-administration of RCAS-PTN enhanced RCAS-PDGF-induced tumor incidence, but did not change the distribution of tumor malignancy grades or tumor histology.

### PTN increases vascular density in PDGF-induced grade III tumors

We have recently shown that PTN expression is associated with increased vascular abnormality and higher vascular area in the GL261 syngeneic orthotopic model of glioblastoma [7]. To investigate if the PTN-induced increase in tumor incidence was associated with its pro-angiogenic effect, we measured the tumor microvessel density and blood vessel diameters. Vessels in grade II tumors where thin-walled and morphologically similar to that of normal brain. No differences in vascular diameter or area were observed when comparing grade II tumors induced by RCAS-PDGFB+RCAS-PTN to grade II tumors induced by RCAS-PDGFB+RCAS-ev (Figure 3C). In contrast, grade III tumors induced by RCAS-PDGFB+RCAS-PTN frequently displayed disorganized, thick-walled vessels not observed in grade III gliomas induced by RCAS-PDGFB+RCAS-ev (Figure 3C). Grade III tumors induced by RCAS-PDGFB+RCAS-PTN also had a significantly higher vascular area and vessel diameter (Figure 3D-3E). This indicates that PTN enhances vascular abnormalization in high-grade tumors, but not low-grade tumors, and that PTN-induced increase in tumor incidence was not generally associated with increased angiogenesis.

### PTN stimulation enhances PDGFB-induced Akt activation in NPCs

PDGFB stimulation initiates tumor formation by stimulation of neural/glial progenitor cells [17]. To investigate mechanisms involved in PTN-induced promotion of PDGFB-induced gliomagenesis, NPCs were treated with recombinant PTN, PDGFB or a combination of PTN and PDGFB. NPC morphology was similar in all treatment groups (Figure S3A).

**Figure 4:**
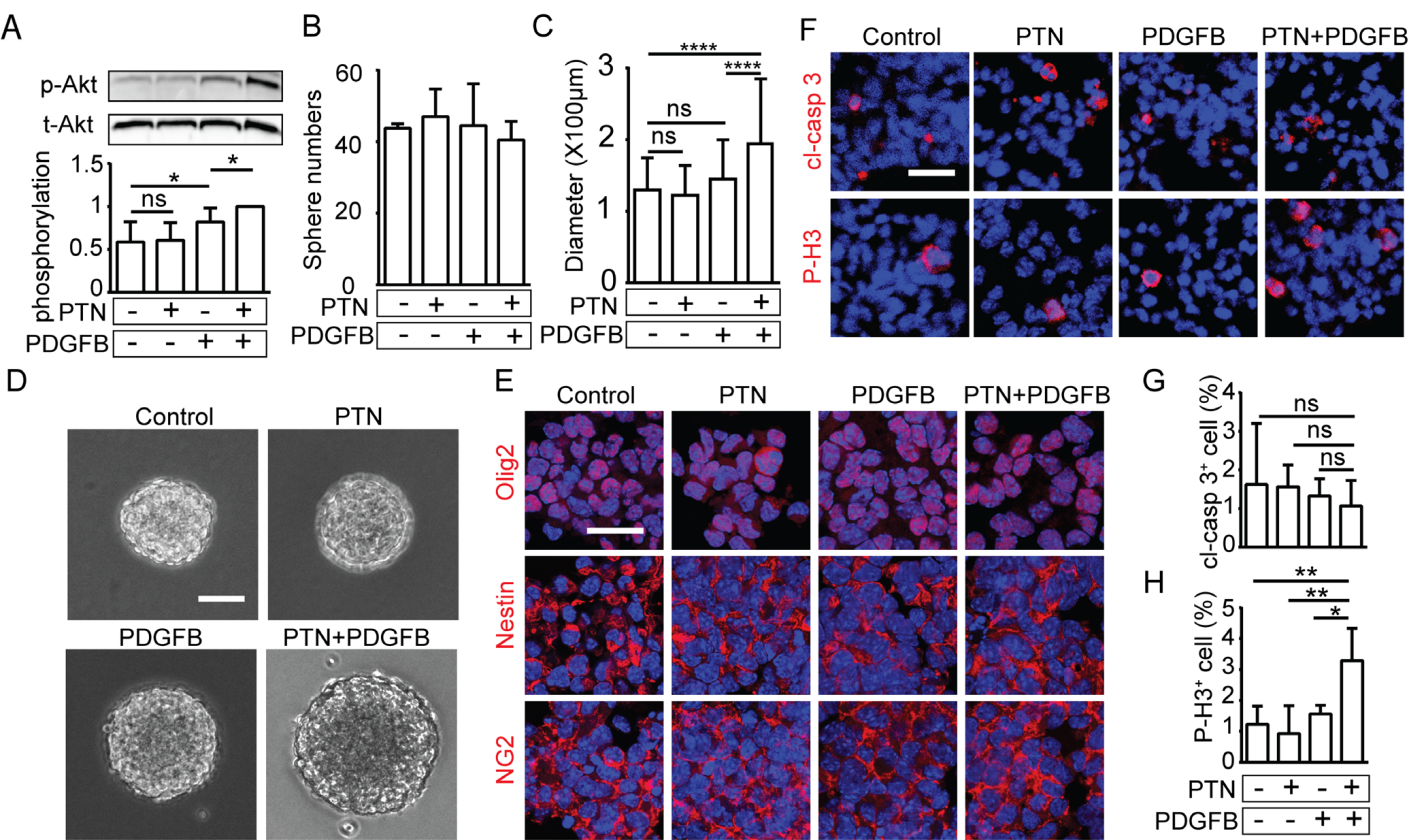
PTN and PDGFB synergistically promote the growth and self-renewal capacity of NPCs **A.** Western blot analyzing phosphorylated (p-Akt) and total Akt (t-Akt) after 4h treatment with PTN, PDGFB or the combination of PTN and PDGFB, and quantification of p-Akt normalized to t-Akt (lower panel), Data is shown as mean ± SD from 7 independent experiments. **B.** Quantification of sphere numbers after stimulation with PTN, PDGFB or PTN in combination with PDGFB. **C.**-**D.** Representative image of spheres upon indicated treatment and quantification of sphere size. (mean ± SD; n ≥ 100 spheres/group). **E.** Immunostaining of olig2, nestin or NG2 in the spheres after the indicated treatment. The experiment was repeated three times. **F.** Immunostaining of cleaved caspase 3 (cl-casp 3) and phosphorylated histone H3 (P-H3) in spheres after treatment, and quantification of **G.** cl-casp 3 and **H.** P-H3 positive cells normalized to total cells (data shown as means ± SD; n ≥ 3 spheres/group). (Bar = 100μm in D, Bar = 20μm in E and F).

Signaling pathways downstream of PDGFR-signaling crucial in PDGFB-induced gliomagenesis were investigated [3]. Src Tyr416 phosphorylation was not induced in response to any treatment regime (Figure S3B and S3D). PDGFB-stimulation increased activation of Erk and Akt in NPCs, whereas PTN-stimulation did not (Figure 4A, Figure S3B-S3C). However, co-treatment with PTN significantly enhanced PDGFB-induced Akt phosphorylation (Figure 4A).

### PTN and PDGFB synergize in promoting growth of neural progenitor cells

To determine if PTN and/or PDGFB affects self-renewal and growth of NPCs, we employed the neural progenitor sphere forming assay. NPCs seeded at clonal density were stimulated with recombinant PTN, PDGFB or PTN in combination with PDGFB. The spheres expressed similar levels of PDGFRA mRNA and PTPRζ mRNA in all treatment groups, while ALK mRNA was undetectable (Figure S4A-S4C).

Sphere numbers were not affected by either treatment, indicating that PTN does not alter the self-renewal capacity of NPCs (Figure 4B). However, sphere size was significantly increased when PTN stimulation was combined with PDGFB (Figure 4C-4D). Sphere size reflects the dynamic status of proliferation in the spheres and the responsiveness to growth factors of the clone-forming cells [18]. The vast majority of cells in all spheres were positive for olig2, nestin and NG2, confirming their identity as neural progenitor cells and indicating that stimulation by PTN and/or PDGFB did not alter their differentiation status (Figure 4E). A similar low percentage of NPCs in the spheres were apoptotic in all treatment groups (Figure 4F-4G). NPC proliferation was significantly increased when spheres were treated by PTN in combination with PDGFB (Figure 4F and 4H). These results demonstrate that PTN and PDGFB synergistically promote proliferation of NPCs, but do not change the differentiation status or the rate of apoptosis.

## DISCUSSION

PTN has previously been suggested to be an oncogene and is highly expressed in glioma, but its role in tumor initiation has not been investigated prior to this report [19]. Our observation that PTN up-regulation in glioma likely occurs through broad amplification of chromosome 7, suggested as one of the earliest steps in gliomagenesis, prompted us to investigate if PTN is involved in transformation of neural progenitor cells, The RCAS/*tv-a* system is designed to induce targeted transformation of neural progenitor cells *in vivo* through viral delivery of oncogenes, allowing us to analyze if constitutive PTN-secretion is sufficient to induce glioma formation. Our results show that PTN-stimulation alone is not sufficient for transformation, even though the experiments were performed in an *Arf*^-/-^ background in which tumor induction is accelerated as compared to wild type mice [16]. However, using *G/tv-a* wild type mice in which gliomas are induced by PDGFB in only roughly a third of the injected mice, we found that PTN markedly enhanced PDGFB-induced tumor formation. PDGF signaling is known to be an initial driving event in glioma evolution, and high level focal amplification of PDGFRA is a feature of the proneural subtype of GBM [3]. Although addition of RCAS-PTN greatly enhanced tumor incidence, it did not change the distribution of tumor malignancy grades or histology of the tumors formed. We have previously demonstrated that PTN induces vascular abnormalization in GL261 orthotopic glioma and that PTN expression correlates with enlarged vascular lumens and abnormal vessel morphology in human glioblastomas [7]. PTN increased vessel area and diameter exclusively in high-grade gliomas, while no difference was seen in low-grade tumors. This indicates that PTN enhances vascular abnormalization in high-grade tumors in combination with other factors in the tumor microenvironment, but that PTN-stimulation is not sufficient to induce vascular abnormalization in low-grade tumors.

Previous work has shown that PDGF stimulation induces tumor formation mainly by transformation of neural/glial progenitor cells [17] [20] [21] [22]. Here, we demonstrate that Erk and Akt are activated in neural progenitor cells upon PDGFB stimulation. Activation of the MAPK pathway by PDGFB has previously been associated with proliferation of glial cells in the brain and tumor initiation [16]. Notably, PTN co-treatment significantly augmented PDGFB-induced Akt phosphorylation. Delivery of constitutively activated Akt is not sufficient to induce glioma formation in the RCAS/*tv-a* model [23]. However, Akt can synergistically enhance cell proliferation and glioma formation induced by MAPK pathway activation by K-Ras [23]. Akt has several important functions, including protecting cell from apoptosis, promoting cell proliferation and maintaining the undifferentiating status of neural and glioma progenitor cells [24] [25]. We found that combined stimulation of neural progenitor cells with PTN and PDGFB significantly increased neural sphere size, but did not change the number of spheres formed. Sphere size is a measure of the dynamics status of proliferation in the spheres [18]. The increase in neural sphere size after co-stimulation with PTN and PDGF was associated with increased proliferation, while the differentiation status and rate of apoptosis was similar to other treatment groups.

PTN/ALK signaling has previously been shown to be required for maintenance of glioma initiating cells [9]. In contrast, we found that murine neural progenitor cells express PTPRz, but not ALK, indicating that the increased Akt-activation and enhanced neural sphere formation is likely due to PTN-induced PTPRz inactivation. Intriguingly, PTPRz dephosphorylates Magi1, a tight junction protein which has been associated with Akt activation through regulation of PTEN signaling [26, 27]. The gene encoding PTPRz is located on chromosome 7 and, consequently, PTPRz up-regulation has been associated with chromosome 7 gain [4]. Our data is consistent with PTN enhancing PDGFB-induced tumor formation through a PTPRz-dependent increase in Akt activation. This work connects chromosome 7 gain, one of the earliest events in gliomagenesis, with increased expression of PTN. PTN is not an oncogene on its own, but potentiates tumor initiation induced by e.g. PDGFB by augmenting Akt activation, leading to enhanced proliferation of neural progenitor cells.

## MATERIALS AND METHODS

### Bioinformatics analysis

To determine the level of PTN expression in different glioblastoma subtypes, patient information and mRNA expression data from glioblastoma samples were collected as described (The Cancer Genome Atlas Research Network 2008). Processed datasets were obtained from the public access data portal (http://www.cbioportal.org/public-portal/). Data was cross-referenced to previously reported subtype classification [2].

The correlation between Chromosome 7 gain and PTN mRNA expression was analyzed using the GlioVis database (http://gliovis.bioinfo.cnio.es/). Gene expression data (HG-U133A for GBM; RNA-seq for LGG) was correlated to gene copy numbers determined by Genomic Identification of Significant Targets in Cancer (GISTC) [15].

Co-expression analysis was performed at cBioPortal database using dataset of Glioblastoma (TCGA, Cell 2013). 41 genes were identified with Pearson's correlation coefficient more than 0.61.

### Production of an RCAS virus to induce autocrine PTN expression

hPTN cDNA was cloned into RCAS-Y [16] to generate RCAS-PTN, correct insertion was confirmed by restriction enzyme mapping and sequencing with BioDye 3.1 (Applied Biosciences). The DF-1 chicken fibroblast cells were cultured as previously described [16]. The RCAS-PTN construct was transfected into the DF-1 cells using Lipofectamine^®^ 2000 transfection agent (11668019, Life technologies).

### Analysis of glioma initiation by RCAS-virus infection in neonatal *G/tv-a* mice

Neonatal *G/tv-a* wt [16] and *G/tv-a;Arf*^-/-^ [16] mice were injected intracerebrally with DF-1 cells producing RCAS-PDGFB [16], RCAS-PTN or RCAS-ev (empty vector) as described [28]. Mice were monitored three times per week and sacrificed either upon any sign of illness or at the endpoint 23 weeks after injection. Mice that died before endpoint were censored in the analysis. The brains were fixed in 4% formalin overnight and embedded in paraffin blocks. Brain tissue was sectioned, H&E stained and analyzed in a blinded procedure by two independent researchers. The tumors were graded based on WHO criteria [1]. To confirm insertion of RCAS-virus in gliomas arising in *G/tv-a* mice, DNA was extracted from paraffin embedded tissue. PCR-detection of gene was done on with specific primers (Table S3) for hPDGFB and hPTN yielding 158bp and 197bp products respectively.

### Immunohistochemical analysis of mouse gliomas

Immunohistochemistry was performed on 5 μm paraffin sections as described [16]. Primary antibodies are listed in Table S4. The images were acquired using the NIS software, stereological quantification for blood vessels was done as previously described [7].

### Western blot

Western blot were performed as described [7]. Antibodies are listed in Table S4.

### cDNA synthesis and qPCR

cDNA synthesis and qPCR were performed as described [7]. Primer sequences are listed in Table S3.

### *In vitro* stimulation of neural progenitor cells

Neural progenitor cells were isolated from *G/tv-a;Arf*^-/-^ mice as described previously [28]. Cells were grown in neural stem cell medium containing DMEM-F12 GlutaMAX (31331093, ThermoFisher), 10mM HEPES (15630049, ThermoFisher ), B27 (12587010, ThermoFisher), penicillin G/streptomycin (P4093,Sigma), 20μg/ml insulin (I-6634, Sigma), 20ng/ml FGF2 (100-18B, Peprotech) and 20ng/ml EGF (AF-100-15, Peprotech). Neural progenitor cells were seeded on 12 well plates at 2x10^4^ per well over night, and then placed in medium without EGF and FGF. After 2 hours, the cells were treated with 25ng/ml PTN (252-PL-050, R&D systems), 10ng/ml PDGFB (100-14b, Peprotech) or the combination of PTN and PDGFB. Cells were harvested 4 hours after treatment.

### Neural sphere forming assay and immunofluorescence staining of spheres

Neural progenitor cells were seeded at 250 cells/ml in neural stem cell medium in ultra-low attachment 6-well plate (CLS3471-24EA, Sigma). 25ng/ml PTN, 10ng/ml PDGF or the combination of PTN and PDGF were added every second day. On day 6, microscopic images of all spheres were taken using a phase contrast microscope to allow size quantification. Then the spheres were fixed with 4% PFA on ice for 10 min, and embedded in OCT medium (Tissue-Tek Sakura) for frozen section. Immunostaining was performed on 7 μm sections. Slides were blocked with 3% bovine serum albumin (Roche Diagnostics) in phosphate-bufferd saline (PBS) and incubated with primary antibody (Table S4) diluted in blocking solution for 2 hours, followed by secondary antibody and nuclear staining with Hoechst 33342 (2μg/ml; Sigma).

### Statistical analysis

Data was analyzed using GraphPad Prism 5.0. The Mann-Whitney test was used for comparison between two groups, and ANOVA with Newman-Keuls test was used for multiple groups’ comparison. Error bars indicate standard deviation from the mean (s.d). Statistical tests were two-sided, and p-values smaller than 0.05 were considered statistically significant.

## SUPPLEMENTARY MATERIAL


