# Supplementary material for: Pleiotrophin enhances PDGFB-induced gliomagenesis through increased proliferation of neural progenitor cells
Source: Oncotarget. 2016 Oct 28;7(49):80382–90. doi: 10.18632/oncotarget.12983 (PMC5348327; doi:10.18632/oncotarget.12983)
Supplement: Supplementary file 1 [file oncotarget-07-80382-s001.pdf]

Pleiotrophin enhances PDGFB-induced gliomagenesis through increased proliferation of neural progenitor cells

Supplementary Material

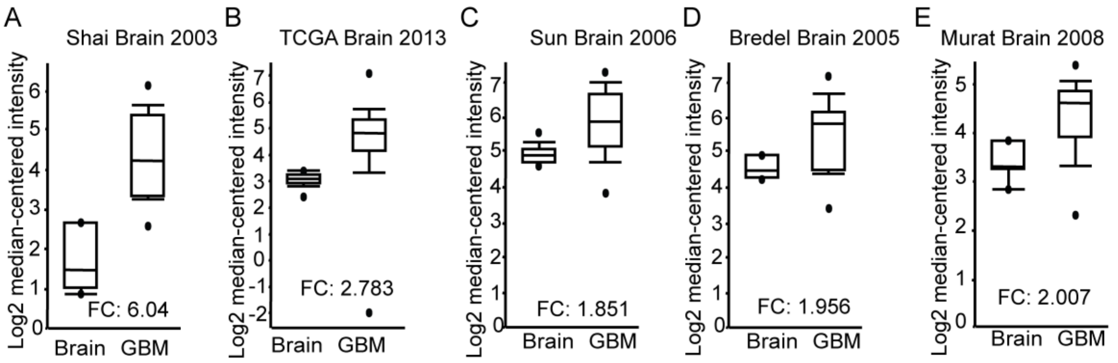

Figure S1. PTN expression is up-regulated in glioblastoma (A-E) Tukey plot showing PTN gene expression levels and fold change (FC) for samples from health brain or glioblastoma patients in the Shai 2003 (A), TCGA 2013 (B), Sun 2006 (C), Bredel 2005 (D) and Murat 2008 (E) datasets obtained from Oncomine.

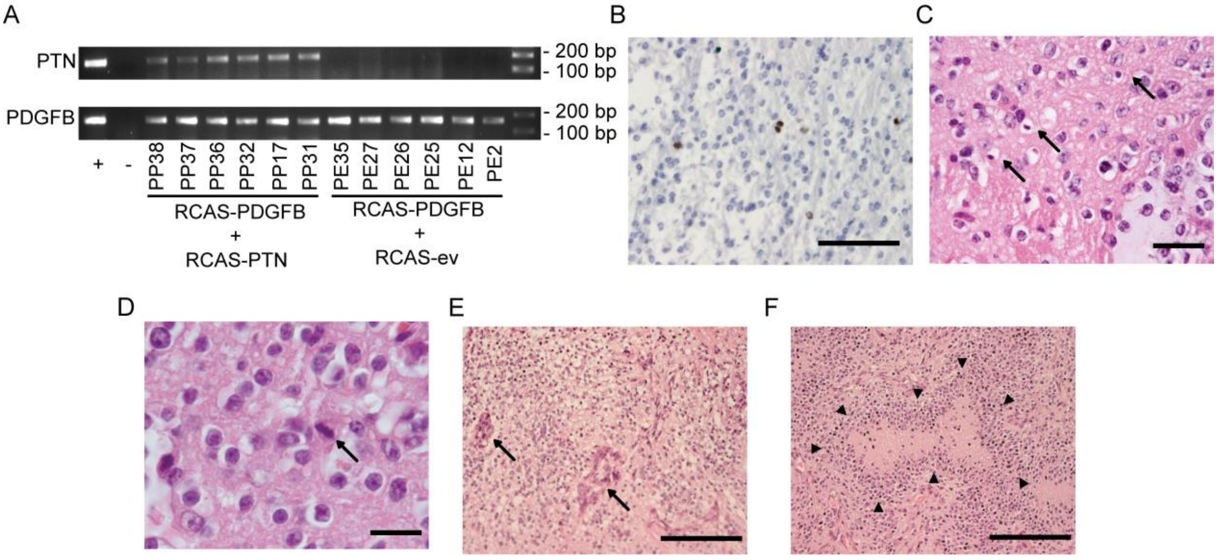

Figure S2. Histopathological features of tumors induced by the RCAS-PDGF virus employed to grade tumors as grade II or grade III. (A) Insertion of the virally transduced PDGF and PTN cDNA in genomic DNA prepared from tumors induced by RCAS-ev+RCAS-PDGF or RCAS-PDGF+RCAS-PTN. (B) Immunohistochemical analysis of the expression Ki-67. (C-F) H&E staining showing apoptosis (C), mitosis (D), microvascular proliferation (E) and pseudopalisading necrosis (F) in the tumor. (Bar = 50 µm in B, Bar = 20 µm in C, Bar = 10µm in D, Bar = 100 µm in E and F)

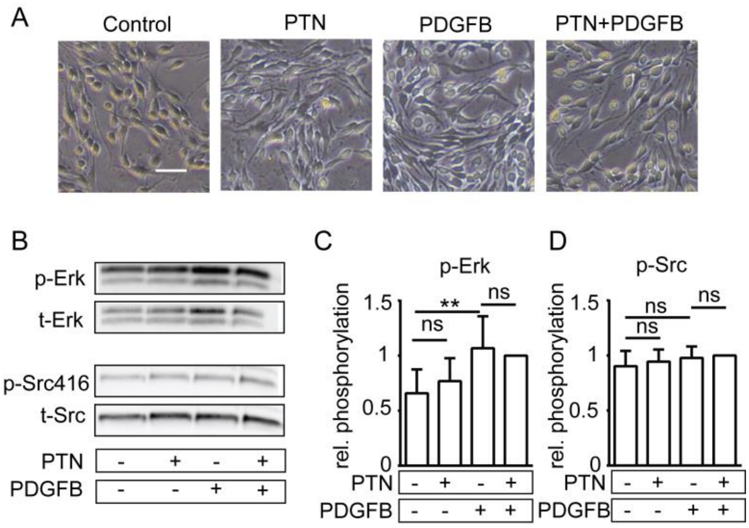

Figure S3. PTN does not affect PDGFB-induced Erk or Src activation in NPCs. (A) Phase-contrast image of neural progenitor cells treated with PTN, PDGFB or the combination of PTN and PDGFB. (B) Western blot analysis showing the levels of phosphorylated (pErk, pSrc) and total Erk (tErk) and Src (tSrc) after 4h treatment with PTN (25ng/ml), PDGFB (20ng/ml) or the combination of PTN (25ng/ml) and PDGFB (20ng/ml). (C-E) Quantification of phosphorylation of Erk (C) and Src (D). Band intensity was normalized to total corresponding protein. Data is shown as mean ± SD from at least 5 independent experiments. (Bar = 50µm)

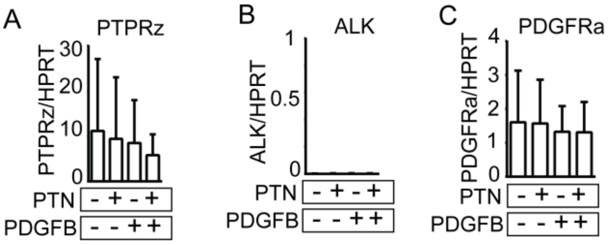

Figure S4. PTPRz and PDGFRα, but not ALK, are expressed in neural spheres, and their expression levels are not changed by stimulation of PTN or PDGFB. (A-C) q-PCR analysis of PTPRz (A) ALK (B) and PDGFRA (C) mRNA expression in neural progenitor spheres treated with PTN (25ng/ml), PDGFB (20ng/ml) or the combination of PTN (25ng/ml) and PDGFB (20ng/ml). mRNA expression levels were normalized to the house-keeping gene HPRT. Data is shown as means ± SD from 3 independent experiments.

Table S1 Overexpression of PTN in Glioblastoma

Dataset	Fold change	Gene rank	Number of samples (normal, glioblastoma)	P value	Meta-analysis
Shai et al.[1]	6.04	298	34(7,24)	3.04E-6	Median rank = 793<br>P=7.46E-13
TCGA	2.783	164	552(10,542)	8.68E-13	
Sun et al. [2]	1.851	793	104(23,81)	7.46E-13	
Bredel et al.[3]	1.956	1724	29(4,25)	7.73E-4	
Murat et al.[4]	2.007	4003	84(4,80)	4E-3	

1. Shai R, Shi T, Kremen TJ, Horvath S, Liau LM, Cloughesy TF, Mischel PS and Nelson SF. Gene expression profiling identifies molecular subtypes of gliomas. Oncogene. 2003; 22(31):4918-4923.
2. Sun L, Hui AM, Su Q, Vortmeyer A, Kotliarov Y, Pastorino S, Passaniti A, Menon J, Walling J, Bailey R, Rosenblum M, Mikkelsen T and Fine HA. Neuronal and glioma-derived stem cell factor induces angiogenesis within the brain. Cancer cell. 2006; 9(4):287-300.
3. Bredel M, Bredel C, Juric D, Harsh GR, Vogel H, Recht LD and Sikic BI. Functional network analysis reveals extended gliomagenesis pathway maps and three novel MYC-interacting genes in human gliomas. Cancer research. 2005; 65(19):8679-8689.
4. Murat A, Migliavacca E, Gorlia T, Lambiv WL, Shay T, Hamou MF, de Tribolet N, Regli L, Wick W, Kouwenhoven MC, Hainfellner JA, Heppner FL, Dietrich PY, Zimmer Y, Cairncross JG, Janzer RC, et al. Stem cell-related "self-renewal" signature and high epidermal growth factor receptor expression associated with resistance to concomitant chemoradiotherapy in glioblastoma. Journal of clinical oncology : official journal of the American Society of Clinical Oncology. 2008; 26(18):3015-3024.

Table S2 Genes co-expressed with PTN in glioblastoma

Gene	Pearson's correlation	Chromosome location
CDK14	**0.7**	Chromosome 7 NC_000007.14 (90596362..91210590)
DENND2A	**0.7**	Chromosome 7 NC_000007.14 (140518420..140641485)
ARL4A	**0.68**	Chromosome 7 NC_000007.14 (12686827..12690934)
CAMK2D	**0.68**	Chromosome 4 NC_000004.12 (113451032..113761927)
CLDN12	**0.68**	Chromosome 7 NC_000007.14 (90403334..90415954)
PDGFA	**0.68**	Chromosome 7 NC_000007.14 (497258..520123)
SPRY2	**0.68**	Chromosome 13 NC_000013.11 (80335976..80340968)
TM6SF2	**0.68**	Chromosome 19 NC_000019.10 (19264365..19273265)
NEK6	**0.67**	Chromosome 9 NC_000009.12 (124257606..124352442)
KBTBD2	**0.66**	Chromosome 7 NC_000007.14 (32868166..32891856)
KCNF1	**0.66**	Chromosome 2 NC_000002.12 (10911937..10914225)
NES	**0.66**	Chromosome 1 NC_000001.11 (156668763..156677397)
ACSL3	**0.65**	Chromosome 2 NC_000002.12 (222861014..222943401)
VAV3	**0.65**	Chromosome 1 NC_000001.11 (107571160..107964923)
DPY19L3	**0.64**	Chromosome 19 NC_000019.10 (32405749..32485893)
FEM1C	**0.64**	Chromosome 5 NC_000005.10 (115520908..115544894)
PCDHGC3	**0.64**	Chromosome 5 NC_000005.10 (141476002..141512977)
PTPRZ1	**0.64**	Chromosome 7 NC_000007.14 (121873105..122062036)
ASPHD2	**0.63**	Chromosome 22 NC_000022.11 (26429314..26445012)
CLIP2	**0.63**	Chromosome 7 NC_000007.14 (74289475..74405943)
ERI1	**0.63**	Chromosome 8 NC_000008.11 (9002804..9033339)
FLOT2	**0.63**	Chromosome 17 NC_000017.11 (28879339..28897697)
LFNG	**0.63**	Chromosome 7 NC_000007.14 (2512529..2529177)
SH3RF1	**0.63**	Chromosome 4 NC_000004.12 (169094256..169271098)
SPRED2	**0.63**	Chromosome 2 NC_000002.12 (65307442..65432637)
TRIM24	**0.63**	Chromosome 7 NC_000007.14 (138460334..138585588)
ZYX	**0.63**	Chromosome 7 NC_000007.14 (143381245..143391111)
CHST12	**0.62**	Chromosome 7 NC_000007.14 (2403560..2434607)
FAM84A	**0.62**	Chromosome 2 NC_000002.12 (14632686..14640046)
METTL7B	**0.62**	Chromosome 12 NC_000012.12 (55681546..55684611)
NAB2	**0.62**	Chromosome 12 NC_000012.12 (57088894..57095476)
NTRK3	**0.62**	Chromosome 15 NC_000015.10 (87876717..88256731)
PTPN9	**0.62**	Chromosome 15 NC_000015.10 (75467121..75579291)
PTPRA	**0.62**	Chromosome 20 NC_000020.11 (2864195..3038669)
CDH2	**0.61**	Chromosome 18 NC_000018.10 (27950966..28177481)
DPF3	**0.61**	Chromosome 14 NC_000014.9 (72616185..72894116)
LMBR1	**0.61**	Chromosome 7, NC_000007.14 (156673354..156893208)
NLGN3	**0.61**	Chromosome X NC_000023.11 (71144831..71171832)
SCRN1	**0.61**	Chromosome 7 NC_000007.14 (29920103..29990289)
SPATA6	**0.61**	Chromosome 1 NC_000001.11 (48260553..48472208)
SPRY4	**0.61**	Chromosome 5 NC_000005.10 (142310427..142325055)

Table S3 PCR primers

	sense	anti-sense
mHPRT	GAAACTTTGCTTTCCCTGGT	TTCGAGAGGTCCTTTTCACC
hPTN (for q-PCR)	GACTGTGGGCTGGGCACACG	TGGTATTTGCACTCCGCGCC
hPTN (for insertion detection)	CCTGGGGAGAATGTGACCTG	CTTTTAATCCAGCATCTTCTCCTG
mPDGFRA	ATGAGAGTGAGATCGAAGGCA	CGGCAAGGTATGATGGCAGAG
mPTPRz	TGCCCAGGGTCCACTGAAGTCC	TGTGACTAGGCGCCCACTGGAT
mALK	CACGTGTGGGGCCAGTGGAC	CCACCTTTCCCGCCAGCTG
hPDGFB	TGCTGCTACCTGCGTCTGGTC	ATGTTCAGGTCCAACTCGGC

Table S4 Antibodies and staining reagents list

Antibody	Company	Catalog No
anti-PTN	Millipore	PL187
anti-Actin	Santa Cruz Biotechnology	sc-1616
anti-Src	Cell Signaling	2123s
anti-Phospho-Src	Cell Signaling	6943s
anti-Erk1/2	Cell Signaling	9102s
anti-Phospho-Erk1/2	Cell Signaling	9101s
rabbit anti-Akt	Cell Signaling	9272s
rabbit anti-Akt (Ser473)	Cell Signaling	9271s
anti-goat- IgG HPR	Sigma-Aldrich	A5420
anti-rabbit IgG HPR	ThermoFisher Scientific	31460
anti-nestin	Covance	PRB315c
anti-olig2	Millpore	AB9610
anti-NG2	Millpore	AB5320
anti-phospho-histone H3	Cell Signaling	9701
anti-cleaved caspase-3	Cell Signaling	9604
anti-rabbit IgG Alexa Fluor 555	ThermoFisher Scientific	A-31572
anti-mouse CD31	Dianova	Dia-310
anti-mouse Ki-67	Dako	M7249
anti-MAP2	abcam	ab32454
anti-GFAP	Dako	Z-0334
biotinylated anti rat IgG antibody	Vector Laboratories	BA9400
biotinylated anti rabbit IgG antibody	Vector Laboratories	BA1000
